# Contribution of Research in the West Indies and Northeast Amazonia to Knowledge of the 2014–2015 Chikungunya Epidemic in the Americas

**DOI:** 10.1007/s40475-021-00242-5

**Published:** 2021-06-19

**Authors:** Timothee Bonifay, Lidvine Godaert, Yanouk Epelboin, Dominique Rousset, Maylis Douine, Hélène Hilderal, Cyril Clavel, Sylvie Abel, Fatiha Najioullah, Laurence Fagour, Margarete do Socorro Mendonça Gomes, Marcus Lacerda, Raymond Cézaire, Narcisse Elenga, Moustapha Dramé, Bruno Hoen, André Cabié, Félix Djossou, Loïc Epelboin

**Affiliations:** 1 Centre d’Investigation Clinique Antilles Guyane, INSERM 1424, Centre Hospitalier de Cayenne, Cayenne, French Guiana; 2 Short-stay Unit, Department of Geriatrics, General Hospital of Valenciennes, Valenciennes, France; 3 Unité d’Entomologie Médicale, Institut Pasteur de la Guyane, French Guiana, Cayenne, France; 4 Virology Laboratory, National Reference Center of Arboviruses, Pastor Institute of Guyana, Cayenne, French Guiana; 5 Infectious Diseases Unit, Centre Hospitalier Louis Constant Fleming, Saint-Martin, France; 6 Department of Infectious Diseases, Centre Hospitalier Universitaire de Martinique, Fort-de-France, Martinique France; 7 Laboratoire de Virologie, Centre Hospitalier Universitaire de Martinique, Fort-de-France, Martinique France; 8 Virology Laboratory, University Hospital of Martinique, Fort de France, France; 9 Laboratório Central de Saúde Pública do Amapá, Macapa, Amapá, Brazil; 10 Fundação de Medicina Tropical Dr. Heitor Viera Dourado, Manaus, Amazonas Brazil; 11 Service de Médecine et Chirurgie Pédiatrique, Centre Hospitalier de Cayenne, Cayenne, French Guiana; 12 Department of Clinical Research and Innovation, University Hospital of Martinique, Fort-de-France, Martinique France; 13 Service de Maladies Infectieuses et Tropicales, Dermatologie, Médecine Interne, Centre Hospitalier Universitaire de Pointe-à-Pitre/Abymes, Pointe-à-Pitre, France; 14 Service des Maladies Infectieuses et Tropicales, Centre Hospitalier de Cayenne, Cayenne, French Guiana

**Keywords:** Chikungunya, Guiana Shield, West Indies, America, Outbreak, Arbovirus

## Abstract

**Purpose of Review:**

Although the chikungunya virus was discovered more than 60 years ago, it has only really been studied since the outbreak in La Reunion in 2005–2006. Ten years later, between 2014 and 2015, the chikungunya virus spread throughout the Americas, affecting millions of people. The objective of this review is to describe the contributions of research on chikungunya virus infection gained from epidemic in the West Indies and the Guiana Shield.

**Recent Findings:**

Prevalence data were similar to those found in the Indian Ocean or Asia during epidemics. Clinically, there is now a better understanding of the typical, atypical, and severe forms. Several studies have insisted on the presence of neurological forms of chikungunya infection, such as encephalitis or Guillain–Barré syndrome. Cases of septic shock due to chikungunya virus as well as thrombotic thrombocytopenic purpura were described for the first time. Given the magnitude of the epidemic and the large number of people affected, this has led to a better description and new classifications of chikungunya virus infections in specific populations such as pregnant women, the elderly, and children. Several studies also described the behavior of populations faced with an emerging disease.

**Summary:**

Current epidemiological data from tropical regions highlights the risk of spreading emerging diseases at higher latitudes, especially concerning arboviruses, since the vector *Aedes albopictus* is already established in many parts of northern countries. A better understanding of the disease and its epidemic dynamics will foster better management, the crucial importance of which was demonstrated during the COVID-19 epidemic.

## Introduction

Chikungunya virus (CHIKV) is an arthropod-borne ARN virus belonging to the Alphavirus genus of the family of Togaviridae that is transmitted by *Aedes sp.* mosquitoes. It was first isolated in 1952 in Tanzania but was never really considered a virus of interest, despite several outbreaks in Asia and Africa, until the Indian Ocean outbreak in 2005–2006 [1]. The outbreak of CHIKV infection (CVI) was particularly well described on La Reunion Island where one-third of the population was suspected to have been infected between December 2005 and June 2006 [2]. With large numbers of cases and focused research, besides common forms, atypical and severe CVI forms were described for the first time [3]. Knowledge acquired during this period considerably amplified the understanding of the pathology. Most of the initially labeled “atypical forms” were in fact common during the unfolding epidemics. In December 2013, the first autochthonous transmission of CHIKV in the western hemisphere was reported on the French part of Saint-Martin, an island in the Lesser Caribbean [4]. Because of the presence of a large immunologically naive population and competent vectors, the outbreak subsequently spread to the other Caribbean islands and the wider Americas, reaching over one million suspected and confirmed cases by December 2014 [5, 6]. Although it continued to circulate in Brazil where 132,205 cases were notified in 2019, this epidemic, despite its large scale, did not result in the establishment of an endemic cycle, like dengue virus or yellow fever virus [7]. The objective of this review was to describe the knowledge gained on chikungunya from the 2014–2015 epidemic in the West Indies and the Guiana Shield.

## Local Epidemiology

**Fig. 1:**
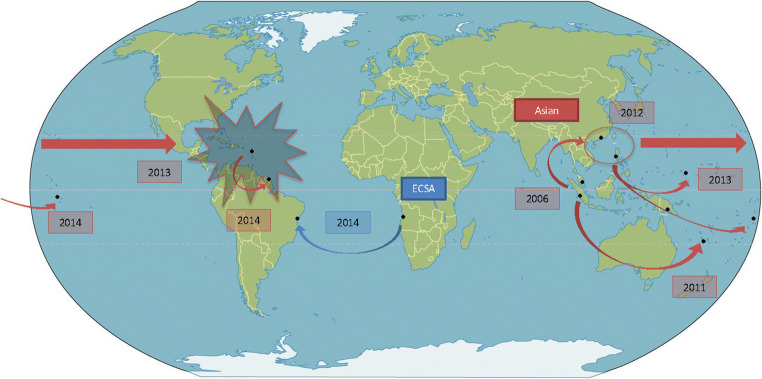
Propagation of the CHIKV epidemic in America in 2013. In red is the Asian strain and in blue the ECSA strain

Virological studies established 4 genotypes of CHIKV: The West African (WAf), East/Central/South African (ECSA), and Asian genotypes, and since 2005 the Indian Ocean Lineage (IOL) which comes from the ECSA one [8]. During the Trinidad epidemic, phylogenetic analyses showed Asian origins to the CHIKV strains were found in the British Virgin Islands at the beginning of the 2013/2014 outbreak in the Americas [9]. Similarly, viruses from Guadeloupe and Martinique were analyzed, confirming the Asian strain origin which was introduced in the French islands from St. Martin [10]. Currently, unless the appearance of mutations makes transmission by *Aedes albopictus* possible, like for the ECSA and IOL strains, the Asian strain only has one vector, *Aedes aegypti*. After 15 years of successive CHIKV epidemics in India and Southeast Asia, the Asian strain has propagated through the East until reaching America, as if competing with the ECSA strain (Fig. 1). Since its discovery in 1952, before the outbreak, there was no known virus circulation in the Caribbean or the Guiana Shield before the outbreak [11]. Two studies, in Suriname, the first in 1963 and, more recently, between 2008 and 2012, did not identify any CHIKV infection [12, 13]. However, because of the epidemic and/or endemic risk, an epidemiological survey would have seemed indispensable to rule out the presence of CHIKV especially in the Amazon area where environmental conditions are optimal for *Aedes* proliferation. Indeed, in Brazil, two distinct strains were notified during the outbreak. Local transmission of the Asian lineage was detected in Brazil for the first time in September 2014 in the Oiapoque municipality, in northern Amapa, on the border with French Guiana. The Asian strain was probably imported to Brazil via this French Overseas territory, which was the first region in America to report CVI. In the same month, a new cluster of CHIKV was notified in the State of Bahia, caused by the ECSA lineage, probably imported from the African coast [14]; indeed, CHIKV appears to be endemic in Angola where thousands of Brazilians, mostly from northeast and southeast areas, work in the petroleum and mining industries [15]. These CHIKV isolates did not contain the A226V or L210Q mutations that are associated with transmissibility by *Ae. albopictus*, suggesting that the CHIKV isolates circulating in Brazil are predominantly transmitted by *Ae. aegypti* [16].

In 2005–2006, the island of La Reunion experienced a large-scale CHIKV epidemic, with rapid dissemination and large numbers of daily patients. CVI seroprevalence levels seemed dependent on the virus circulation mode. Seroprevalence was around 40% for epidemic modes (38.2% prevalence in La Reunion [2], 37.2% in Mayotte [17], or 34% in Kenya [18], and it was much higher in endemic transmission, like in Lamu where 72% of the population was positive for CHIKV [19]. In the Americas, the incidence curves were variable—an abrupt epidemic peak in Guadeloupe and a flatter epidemic growth in Martinique and French Guiana for example [20]. In Saint Martin Island, in July 2014 during the outbreak, and 7 months after its introduction, the CHIKV seroprevalence was 16.9% in the population and 39.0% of infections remained asymptomatic [21]. In the French West Indies, in blood donors from Guadeloupe and Martinique, CHIKV seroprevalence was 48.1% and 41.9%, respectively, in January 2015 [20]. At the end of the outbreak, 377 patients living with HIV (PLHIV) followed in Martinique and Guadeloupe were randomly selected to match the age and sex distribution of the general population in the two islands. CHIKV serology was positive in 230 patients, which represented a seroprevalence rate of 61% (95% CI 56–66), with only 153 patients reporting symptoms consistent with CVI [22]. In French Guiana in 2017, a sero-epidemiology study found a 20.3% prevalence (18.5–22.1%), with 45.2% of asymptomatic infections [23]. Another study in Puerto Rico demonstrated that nearly 25% of blood donors acquired CHIKV infections and seroconverted during the epidemic [24]. These seroprevalence studies have shown highly heterogeneous results in part reflecting the heterogeneity of study designs and serological tests used, but also differences in human and vectorial factors [25].

## Vector Competence Evidence

Entomological studies related to the 2013–2014 chikungunya epidemic in the Americas focused on 3 main themes: vector competence (the intrinsic ability of mosquitoes to be infected, multiply, and transmit a pathogen to another host), the presence or absence of CHIKV in mosquitoes collected in the field, and their resistance to insecticides, which is directly linked to interventions to avoid the spread of the epidemic. In 2011, a first study with *Ae. aegypti* populations from French Guiana and the French West Indies revealed their high competence to transmit CHIKV [26]. Other entomological studies also showed the high competence of several populations of *Ae. aegypti* and *Ae. albopictus* species to transmit three different CHIKV genotypes [27, 28]. Moreover, in laboratory conditions, exposure of European *Ae*. *albopictus* to low temperatures (20 °C) significantly reduced the transmission of CHIKV strains from the Americas, suggesting that colder temperatures may decrease the local transmission of CHIKV by European *Ae*. *Albopictus* [28]*.* Two studies reinforced the idea that *Ae. aegypti* was the main vector during CHIKV epidemics by detecting the virus in field-collected females, in Martinique and in French Guiana [26, 29], but virus detection in field mosquitoes remained quite low.

In the French overseas territories and in Europe, deltamethrin, a pyrethroid compound, is the only allowed molecule to decrease adult mosquito populations. However, in 2014 during the CHIKV epidemic, malathion, an organophosphate compound, was exceptionally authorized for 6 months in French Guiana. However, the lack of evidence of its effectiveness in containing the epidemic, the mistrust from the population against this chemical, and data released on the cancerogenic effects of this molecule pushed local authorities to halt its use [30]. Hence, because *Ae. aegypti* populations from French West Indies and French Guiana exhibit multiple resistances to organophosphates (temephos and malathion), and pyrethroids (deltamethrin), the effectiveness of vector control is compromised [31–33].

## Clinical Forms

### Clinical Presentations and Classifications

Knowledge acquired during the 2005–2006 period improved our understanding of the disease. Indeed, CVI was hardly known before the Indian Ocean epidemics. The outbreak in La Reunion Island led to the accumulation of knowledge about this infection and especially about new clinical forms. CHIKV was known to cause multiple joint pain and fever, but new forms, besides severe forms, called atypical forms, became increasingly reported. Most of the initially labeled “atypical forms,” for example dermatological forms with a rash and polymorphous eruption, became considered “common forms” during the following epidemics. In 2015, the World Health Organization (WHO) assembled an expert group to develop consensus definitions of the clinical forms of CVI. The resulting definitions described three clinical forms at the acute phase, based on clinical, epidemiological, and laboratory criteria [34•]. (1) A confirmed typical case is defined by “fever AND joint pain with acute onset” AND “residing or visiting areas with local transmission of Chikungunya” OR “laboratory confirmation by immunoglobulin or RT-PCR”. Most of the time, adult patients had a classical form defined by a febrile polyalgic syndrome with predominantly distal joint pain and a rash with 2 stages—early rash followed by secondary polymorphous eruptions—with diffuse pruritus and fluctuating digestive signs [35, 36]. (2) Confirmed atypical cases are defined by the same criteria AND the presence of other clinical or biological manifestations—including neurological, cardiovascular, and liver anomalies. (3) Confirmed severe cases are defined by the same criteria and dysfunction of at least one organ or system that threatens life and requires hospitalization [37].

### Neurological Forms

As for several members of the alphavirus family, CHIKV have a strong neurological tropism. Most persons with neurological forms had encephalitis or myelitis. During the outbreak, the incidence of Guillain–Barré syndrome (GBS) was greater than usual, and for the first time, the association between CVI and GBS was shown [38••]. In Martinique and Guadeloupe, 13 cases of CVI-related GBS were identified in 10 men and 3 women. The calculated annual incidence rate of GBS in the French West Indies’ general population was 3.45/100,000 inhabitants in 2014, compared with 1.77/100,000 inhabitants over the 2011–2013 period, a significant 2-fold increase during the year of the chikungunya outbreak (P = 0.006) [39]. In French Guiana, a study conducted at Cayenne hospital showed that the more frequent atypical form was neurological, with several cases of GBS, encephalitis, hydrocephaly, and stroke [40]. CHIKV seems to have a substantial negative impact on patients with preexisting neurological disorders, such as parkinsonism, multiple sclerosis, and sequelae of hemiplegia [41].

### Description of New Atypical or Severe Forms

In Guadeloupe, 450 patients with confirmed CVI were admitted to the University Hospital of Pointe-à-Pitre. Of these, 25 had severe sepsis or septic shock and 12 died. This finding strongly suggested that CHIKV can, in rare cases, cause severe sepsis and septic shock syndromes, without any bacterial co-infection [42]. It is of note that the patients who presented severe forms did not present more comorbidities or were not older than those who presented classic or atypical forms. Hematological manifestations were also described. The first documented case of thrombotic thrombocytopenic purpura (TTP) associated to CHIKV was described in French Guiana [43]. PTT is a thrombotic microangiopathy associated to severe ADAMTS 13 deficiency. It has been linked to various viral infections. Among arboviruses, only Crimean–Congo hemorrhagic fever and dengue fever have been linked to this severe disease.

### Hospitalizations and Mortality due to Chikungunya Virus Outbreak

In French Guiana, during the outbreak, most hospitalizations were common forms, driven by painful clinical presentations, and concerns due to the novelty of this infection [40]. As for many other infectious diseases, a very high prevalence of decompensations of underlying diseases was observed, and explained a large part of hospitalizations. The true magnitude of mortality in the French Caribbean islands may have escaped traditional surveillance systems [44]. However, calculating excess deaths—the difference between the expected and observed deaths for all age groups for each month in 2014 and 2015—showed the peak of mortality coincided with the epidemic peak and returned to normal soon after the end of the CHIKV epidemic. A correlation between monthly excess deaths and reported cases of chikungunya was shown both for monthly rates of hospitalization for CHIKV and for excess deaths with a delay of 1 month. There were excess deaths in almost all age groups, especially in the elderly group. The overall mortality estimated in the study (639 deaths) was about four times greater than that obtained through death certificates (160 deaths). However, in French Guiana, despite atypical neurological and liver forms of CVI, case-fatality was low [40]. The younger population with fewer underlying medical complications may have explained the lower mortality.

### Differential Diagnoses

In tropical areas, a large number of arboviral or infectious diseases coexist, such as dengue virus (DENV), Mayaro virus (MAYV), leptospirosis or malaria, which may be difficult to diagnose, especially without biological tests [45, 46]. Because of non-specific clinical presentation, a predictive score was developed in French Guiana to differentiate CVI to DENV infection at the acute stage of the disease. Over the study period, 168 patients infected with CHIKV were compared with 452 patients with DENV. The clinical variables independently associated with CHIKV were joint and back pain, and those associated with DENV were headache, muscle pain, nausea/vomiting, diarrhea, and hemorrhagic signs. The clinical score had 98% sensitivity for DENV and the area under the ROC curve was 0.96 [36]. A study in 660 children hospitalized in general pediatric wards found 106 patients with malaria, dengue, or chikungunya during a 2-year follow-up (2012 and 2015). In multivariate analysis, patients with malaria had the highest frequency of hepatomegaly and C-reactive protein elevation, whereas patients with chikungunya had the highest pain level and irritability. Hepatomegaly was also frequent in dengue [47]. No study comparing MAYV infection or Tonate virus to CVI was conducted. Both are arboviruses from the alphavirus genus, and their infection can lead to CHIKV-like or DENV-like syndrome, which can lead to misdiagnosis [48, 49].

### Chronic Post-chikungunya Rheumatic Disease

Chronic rheumatic complications after acute CVI were frequent. The term “chronic chikungunya syndrome” covers multiple etiologies, including reactivation of osteoarthritis and posttraumatic degenerative manifestations, fibromyalgia, spondylo-arthritis, and de novo polyarthritis. In Martinique, rheumatologists identified 13% of misdiagnosed patients, which conducted to late treatment initiation. They also noted that 21.1% of patients progressed to a chronic form of de novo seronegative rheumatoid arthritis requiring intensive therapy [50]. Several studies were conducted to determine the prevalence of chronic symptoms. In Martinique, 200 of 509 participants (39.3%) still declared feeling symptoms, associated with the probable chronic stage CVI, 3 months or more after the initial acute phase [51, 52]. Also in Martinique, another study in 167 participants revealed a 52% prevalence of chronic chikungunya arthritis at 12 months [53]. In French Guiana, at 3 months, 40.2% (n = 45/112) still had clinical manifestations and 31.3% (n = 31/99) still did at 6 months. The median time of pain disappearance was 2 weeks after the onset of signs [54].

## Specific Populations

From the beginning of the outbreak, physicians and authors reported that the definition of the clinical forms of CVI was not applicable to infants and the elderly [55]. Pediatric and geriatric specificities were hence studied during the epidemics.

### Elderly People and CVI

A study by Godaert et al. suggests that clinical presentations of the acute phase of CVI in the elderly differ from that in younger adults [56]. The elderly population were more likely to have atypical form of the disease than their younger counterparts (29.6% vs. 5.6%; p < 0.001). Moreover, the majority of the elderly (42.7%) presented a clinical form of CVI that was not captured by the acute phase WHO 2015 classification, with absence of fever, or absence of joint pain, or both [56]. New findings suggest that the definition of the clinical forms of CHIKV at the acute phase must be adapted in elderly subjects. The authors therefore propose to replace the pair “fever and arthralgia” of the 2015 WHO definition with “fever and/or arthralgia,” because many older people do not experience both signs at the beginning of CHIKV infection. It is thus of great importance to adapt the definition in order to make it easier for physicians to diagnose CHIKV in older populations during outbreaks [57]. To help diagnosis in this specific population, a score was developed from four variables independently associated with positive RT-PCR: fever (3 points), pain of the ankles (2 points), lymphopenia <1000/mL (6 points), absence of elevated polynuclear neutrophil count (below 7500/mL) (10 points). The best cut-off of the score was ≥12 with 87% (83–90%) of sensitivity and 70% (63–76%) specificity [58•]. The use of specific diagnostic scores and appropriate definition for the elderly population could reduce the risk of misdiagnosis [59].

### Children and CVI

The epidemic affected a third of the population in Guadeloupe in just a few months, including children. These studies highlighted a clinical presentation of pediatric CVI combining pain, fever, rash, and edema of the extremities. A study in Guadeloupe, including <24 months old infants, supported these findings; more than 80% of the infants had fever, 69% had a rash, and 48% had hands and/or feet edema [60]. Half of the infants presented edema of the extremities. The presence of cutaneous signs was more frequent in pediatric and neonatal forms than in adults. In French Guiana, a study about infants younger than 3 months showed the most important clinical findings were fever >39 °C (81%) for 48 h or more (77%), irritability (96%), and skin rash (69%) [61]. It is important to note a case of pediatric septic shock, similar to the one described in adults, reported in Suriname in a 13-year-old child with the resolution of symptoms after rehydration and pain medication [62].

### Pregnancy and CVI

During the epidemic in La Réunion island in 2005/2006, there were frequent and serious neonatal forms of infection: neurosensory, neuropsychological, or neurological disorders such as epilepsy or cerebral palsy (52.6%). To try to prevent these complications, a phase I/II open-label, non-randomized, multicenter therapeutic trial conducted by Guadeloupe’s university hospital was conducted in 4 centers in the Antilles-French Guiana region during the 2014 CHIKV epidemic. The objective was to evaluate the tolerance and safety of intravenous administration of anti-CHIKV human immunoglobulins to newborns at risk of mother-to-child transmission (MTCT) of CHIKV (newborn of a viremic mother at the time of delivery or within 48 h post-delivery). These anti-CHIKV immunoglobulins were developed from CVI-convalescent plasma donors. In French Guiana, only one newborn baby was injected with immunoglobulins; two others were eligible but the parents refused. Unfortunately, the necessary number of inclusions was never reached due to the strict inclusion criteria, and the original question was never answered. A multi-centric trial including more countries in the region could be considered in the event of a new outbreak of CHIKV to increase the sample size and allow concluding on the efficacy of these immunoglobulins, given the potential severity of maternal–fetal infections. Parallel to the CHIKIVIG study, 15 MTCT cases were reported, 5 in Martinique and 10 in Guadeloupe. Seven MTCT cases developed serious clinical manifestations, 4 of which presented cardiovascular failure but no death was reported [63]. A study was conducted in Curaçao and described the outcomes of symptomatic neonates with vertically transmitted CHIKV infection during the CHIKV epidemic [64]. There were three symptomatic neonates with serologically confirmed infection out of 61 pregnant women. Two neonates developed neurological complications, including convulsions and intracerebral bleeding. One newborn, for whom maternal infection occurred 7 weeks before delivery, died after birth. As known during the La Reunion outbreak, these results confirmed the potential severity of maternal–fetal transmission of CHIKV. There was hence a widespread need to share experiences and to implement protocols for the management of perinatal CHIKV infection. These recommendations were relevant and applied during the Zika virus outbreak of 2015–2016.

### Patient Living with HIV (PLHIV)

During the CHIKV outbreak and the 6 following months, of 1003 PLHIV in care in the center of infectious diseases in Martinique, 188 (94 men and 94 women) had confirmed CVI. Clinical presentation was common in 63% of the cases, and severe and atypical forms were scarce. During the acute phase, CD4+ and CD8+ T-cell (evaluated in 30 PLHIV, 15 men and 15 women) absolute numbers dropped significantly, but returned to pre-CHIKV values after the acute phase. Reassuringly, CD4 and CD8 T cell proportions did not decrease during the acute phase. CHIKV infection had no significant impact on this anti-retroviral-treated population [65].

### Poverty and CVI

In view of the current importance of neglected tropical diseases, its impact on different populations has been studied, in comparison with dengue. The present results suggest that early in the epidemic, the populations most at risk for CHIKV infection were the most socially vulnerable populations in the poorest neighborhoods, whereas DENV appeared to have affected a richer population and richer areas [66•]. Similar findings were found during Zika’s outbreak in French Guiana [67]. Further investigations are required to disentangle the potential determinants of this observation.

## Vaccination

A clinical trial was conducted at 6 clinical research sites located in Haiti, Dominican Republic, Martinique, Guadeloupe, and Puerto Rico, to evaluate the safety and tolerability of an investigational CHIKV-like particle (VLP) vaccine in endemic regions. This was a randomized, placebo-controlled, double-blind, phase 2 clinical trial to assess the vaccine VRC-CHKVLP059-00-VP (CHIKV VLP). A total of 400 healthy adults aged 18 through 60 years were enrolled between November 2015 and March 6, 2018. Of the 400 participants (mean age, 35 years; 199 [50%] women), 393 (98%) completed the primary safety analysis. All injections were well tolerated. Of the 16 serious adverse events unrelated to the study drugs, 4 (25%) occurred among 4 patients in the vaccine group and 12 (75%) occurred among 11 patients in the placebo group. The durability of the immune response was demonstrated through 72 weeks after vaccination. Phase 3 trials are now needed to assess clinical efficacy [68].

## Reflections About Behavior and Its Dynamics

Several million people were affected by CHIKV as it spread in more than 90% of American countries. That led to many behavior and dynamic studies of these populations. An interesting study performed in French Guiana about student perception of CHIKV at the beginning of the outbreak showed that CVI was associated with the highest scores in terms of perceived severity, or fear of its consequences (comparing it to other diseases like dengue, yellow fever, or malaria). This score then dropped once the virus was in circulation because of a context of emergence of a new virus. These results also revealed that, unsurprisingly, the adoption of protective behaviors is a multi-factorial process that depends on both sociocultural and cognitive factors [69, 70]. Because of these factors, more local studies are needed for adapting health authorities’ responses. An additional longitudinal study showed that (1) the frequency of some health behaviors significantly increased with the subjective and objective prevalence of the disease, (2) perceived risk of infection for oneself tended to decrease considerably over time, and (3) the risk reappraisal hypothesis failed to account for this paradoxical trend in the people's response to the risk of contracting the disease [71]. These reflections could be extended to other infectious disease epidemics, such as COVID-19, and also lead to changes in the type of intervention (personal versus environmental methods) during the outbreak. A dynamic identification of clusters can lead to local risk assessment and provides opportunities for targeted control efforts for nations experiencing CHIKV outbreaks [72].

## Conclusion

Since 2013, the Guiana Shield and the West Indies have seen 4 different arboviral outbreaks: DENV, CHIKV, Zika virus, and yellow fever virus, not counting Covid-19. All of these diseases were public health issues. Several risks should be kept in mind. Mutations led to potential CHIKV transmission by *Aedes albopictus*, which is present at higher latitudes all around the world and is already responsible for outbreaks, such as CHIKV in India. Due to important human fluxes between America and Europe, and to the increasing presence of *Aedes albopictus* in the old continent, there is a non-negligible risk of diffusion of these arboviruses in Western Europe. Although Europe has been, until recently, spared by the phenomenon, it has been reported as suitable for dengue and chikungunya for some years now [73, 74]. Tropical and equatorial areas are frequently impacted by arbovirus outbreaks; there is therefore a need to study other arboviruses, autochthonous ones such as Mayaro virus, Tonate virus, Oropouche virus, or O’Nyong Nyong viruses which could be involved in future epidemics in Latin America and the Caribbean.
